# Why Age Matters: The Importance of Historical Context in the Study of Minority Stress in Sexual Minorities

**DOI:** 10.1093/geroni/igaa057.2341

**Published:** 2020-12-16

**Authors:** Michael Vale, Toni Bisconti

**Affiliations:** 1 University of Akron, Akron, Ohio, United States; 2 Univesity of Akron, Akron, Ohio, United States

## Abstract

Currently, there is support that links experiences of stigma/discrimination, known as minority stress, with depressive symptomatology in older sexual minorities (OSM). Yet, the context of cohort is ignored, despite OSM having been exposed to greater stigma across their lifetime. The current project explores how cohort informs the minority stress process by comparing three minority stressors and their relationships with depressive symptomatology across a younger (18-39, N=129) and older sample (40-80, N=104). It was found that OSM had significantly less overall stress, had different significant links between each stressor and depressive symptomatology, and significantly used social support to buffer stress contrasting their younger counterparts. These results highlight the need to consider cohort and discover why OSM are more resilient to minority stress. Considering the role of cohort improves aging and minority stress theories, is useful for professionals improving well-being in OSM, and informs our understanding of the lived experiences of OSM. Part of a symposium sponsored by Rainbow Research Group Interest Group.

